# SGLT2 inhibition with empagliflozin attenuates myocardial oxidative stress and fibrosis in diabetic mice heart

**DOI:** 10.1186/s12933-019-0816-2

**Published:** 2019-02-02

**Authors:** Chenguang Li, Jie Zhang, Mei Xue, Xiaoyu Li, Fei Han, Xiangyang Liu, Linxin Xu, Yunhong Lu, Ying Cheng, Ting Li, Xiaochen Yu, Bei Sun, Liming Chen

**Affiliations:** NHC Key Laboratory of Hormones and Development (Tianjin Medical University), Tianjin Key Laboratory of Metabolic Diseases, Tianjin Medical University Metabolic Diseases Hospital & Tianjin Institute of Endocrinology, Tianjin, 300070 China

**Keywords:** Type 2 diabetes mellitus, SGLT2, Empagliflozin, Oxidative stress, Myocardial fibrosis

## Abstract

**Background:**

Hyperglycaemia associated with myocardial oxidative stress and fibrosis is the main cause of diabetic cardiomyopathy. Empagliflozin, a sodium-glucose cotransporter 2 (SGLT2) inhibitor has recently been reported to improve glycaemic control in patients with type 2 diabetes in an insulin-independent manner. The aim of this study was to investigate the effect of empagliflozin on myocardium injury and the potential mechanism in type 2 diabetic KK-Ay mice.

**Methods:**

Thirty diabetic KK-Ay mice were administered empagliflozin (10 mg/kg/day) by oral gavage daily for 8 weeks. After 8 weeks, heart structure and function were evaluated by echocardiography. Oxidants and antioxidants were measured and cardiac fibrosis was analysed using immunohistochemistry, Masson’s trichrome stain and Western blot.

**Results:**

Results showed that empagliflozin improved diabetic myocardial structure and function, decreased myocardial oxidative stress and ameliorated myocardial fibrosis. Further study indicated that empagliflozin suppressed oxidative stress and fibrosis through inhibition of the transforming growth factor β/Smad pathway and activation of Nrf2/ARE signaling.

**Conclusions:**

Glycaemic control with empagliflozin significantly ameliorated myocardial oxidative stress injury and cardiac fibrosis in diabetic mice. Taken together, these results indicate that the empagliflozin is a promising agent for the prevention and treatment of diabetic cardiomyopathy.

## Introduction

The global incidence of diabetes mellitus (DM) has gradually increased over the past three decades and has become a major public health problem worldwide [1]. Global estimates of diabetes prevalence in 2017 indicate that 451 million people aged 18–99 years are affected with diabetes worldwide, and 693 million people are projected to have diabetes by the year 2045, with most individuals suffering from type 2 DM [2]. Uncontrolled diabetes can lead to a number of short- and long-term complications, including heart disease, dyslipidaemia, diabetic nephropathy and nerve damage, and vision problems. Cardiovascular disease is the leading cause of death among all of the diabetic complications [3]. Diabetic cardiomyopathy is defined as a diffuse myocardial fibrosis, myofibrillar hypertrophy, and impaired contractile function in the absence of valvular, hypertensive, or ischemic heart disease [4, 5]. However, despite the numerous experimental and preclinical studies that have been conducted, the precise mechanisms underlying the pathogenesis of diabetic cardiomyopathy are still uncertain.

Oxidative stress is known to be associated with the development of diabetes. A growing body of evidence suggests an increase in oxidative stress in response to hyperglycaemia in vascular tissues of patients with DM [6, 7]. Several reports have shown that an increased level of reactive oxygen species (ROS), induced by hyperglycaemia, leads to impaired contractile function and myocardial fibrosis in the left ventricle of diabetic rats [8, 9]. In addition, the extent of myocardial fibrosis is associated with impairment in cardiac contractile function. ROS directly induces DNA damage and activates poly (ADP-ribose) polymerase (PARP-1) in the nucleus, which results in the depletion of cellular nicotinamide adenine dinucleotide (NAD^+^) and adenosine triphosphate, mitochondrial permeability transition pore opening, mitochondrial dysfunction, and initiation of cell death via both necrosis and apoptosis [10]. Further ROS can promote myocardial fibrosis by producing profibrotic factors, such as transforming growth factor β (TGF-β) and activating the epithelial mesenchymal transition process of the differentiation of cardiac fibroblasts into extracellular matrix producing myofibroblasts [11]. Moreover, ROS causes the degeneration of lipid, protein, and nuclei acid and exacerbates the cardiac inflammatory response and cardiomyocytes apoptosis. Therefore, to prevent the development of diabetic cardiomyopathy, more effective therapies should be used both to manage blood glucose to near-normal levels and reduce oxidative stress. However, research on the effects of antioxidants on cardiac fibrosis and remodelling remains scarce.

Empagliflozin, a sodium-glucose cotransporter 2 (SGLT2) inhibitor, is a newly developed oral antidiabetic drug to enhance renal glucose excretion or glycosuria and reduce hyperglycaemia in an insulin-independent manner by highly selective inhibition of SGLT2 [12]. SGLT2 is mainly located in the apical brush border membrane of the S1 segment of the proximal convoluted tubules, which regulates 90% of the reabsorption of glucose from glomerular filtrate. Therefore, SGLT2 inhibitor can increase the urinary glucose level and reduce blood glucose. Empagliflozin is different form conventional antidiabetic drugs, which rely on insulin secretion, and represents a novel class of antidiabetic drugs. It has been approved for the treatment of type 2 diabetes in adults since 2014. Studies performed in streptozotocin-induced type 1 diabetic rats have shown that empagliflozin can reduces ROS in pancreatic β-cells [13]. Another study revealed that empagliflozin can ameliorate oxidative stress in aortic vessels and prevent endothelial dysfunction in the aortic rings of streptozotocin-induced type 1 diabetic rats [14]. However, little is known about whether empagliflozin can reduce the oxidative stress of cardiac tissue and prevent cardiac fibrosis and remodelling in type 2 diabetic patients.

In the current study, we assess the efficacy of the SGLT2 inhibitor empagliflozin in reducing oxidative stress and preventing diabetic-induced myocardial fibrosis using the type 2 diabetic KK-Ay mice model. We also explore the downstream signalling of myocardial fibrosis activated by oxidative stress in the left ventricle of a type 2 diabetic mice model. We further evaluate its effect on left ventricular dysfunction and myocardial structural damage induced by type 2 diabetes.

## Methods

### Animal procedure and drug treatment

Thirty 8-week old KK-Ay mice (genetic type 2 diabetes model) and C57BL/6J mice were purchased from Beijing HFK Bioscience Co, Ltd (Beijing, China). The procedures of the experiments were authorized and specifically approved by the institutional ethical committee of Tianjin Medical University. Mice were housed in cages (4–6 per cage) with free access to the drink/feed boxes. Mice were housed in a room kept at 24 °C with 12:12 h light/dark cycle.

The type 2 diabetes model mice were fed a high-fat diet. Blood glucose was measured daily. The mice with a blood glucose concentration greater than 15 mM (200 mg/dL) for 2 consecutive weeks were used for the following experiments. Subsequently, animals were randomized into groups (15 per group) and raised for 8 weeks. The groups were as follows: (1) control group: healthy C57BL/6J mice with no treatment; (2) DM group: type 2 diabetic KK-Ay mice with no treatment; (3) DM + empagliflozin (DM + EM) group: type 2 diabetic KK-Ay mice treated with empagliflozin (10 mg/kg/day for 10 weeks, oral gavage, 0.5% hydroxyethylcellulose was used as the vehicle). Empagliflozin, a selective SGLT2 inhibitor, was provided by Boehringer Ingelheim Pharma GmbH & Co. KG (Germany).

### Echocardiographic evaluation

Echocardiographic measurement was performed before the experimental intervention and at the end of the study period. We used M-mode echocardiography equipped with 17.5 MHz liner array transducer system (Vevo 2100™ High Resolution Imaging System; Visual Sonics). As described in a previous study [15], the heart rate (HR) and the following structural variables were evaluated: left ventricular internal dimension in diastole (LVIDD), left ventricular internal dimension in systole (LVIDS), interventricular septal thickness in systole (IVSs) and in diastole (IVSd), and LV posterior wall thickness in systole (LVPWs) and diastole (LVPWd). LV mass was calculated using the formula [(LVIDd + LVPWd + IVSd)^3^ − (LVIDd)^3^ × 1.04 × 0.8 + 0.6]. LV function was assessed by the following parameters including fractional shortening (FS), ejection fraction (EF), and E/A ratio. All measurements were conducted by a single investigator who was blinded to the experimental groups.

### Myocardial hydroxyproline concentration

The myocardial hydroxyproline concentration of the left ventricle was measured to estimate the myocardial collagen content according to the methods described by Colgrave [16]. The measurements were performed by a spectrophotometer using commercial kit (BioVision, Mountain View, CA, USA) according to the manufacturer’s protocols. The results were expressed as ng/mg total protein.

### Detection of serum lipids, glucose, insulin, and HbA1c levels

At the end of the study, mice were anaesthetized by inhalation of 3% isopentane in air. The fasting blood specimens were collected into commercial tubes containing lithium heparin as an anticoagulant via the sublingual vein from each animal and centrifuged for 6 min at 3000 rpm/min. The plasma was kept in a plain tube and stored at − 20 °C until analysis. Total cholesterol (TC), triglyceride (TG), low-density lipoprotein cholesterol (LDL-C), and high-density lipoprotein cholesterol (HDL-C) plasma concentrations were determined by spectrophotometric methods. Blood glucose levels were measured using a glucometer (ACCU-CHEK, Roche, USA). After an overnight fast, fasting insulin and proinsulin levels were determined using mouse insulin and proinsulin enzyme-linked immunosorbent assay (ELISA) kits, respectively (Nanjing Jiancheng Biotech, China). Blood was also retained for measurement of HbA1c by a chromatographic–spectrophotometric-ion exchange kit (Biosystems, Spain).

### Assessment of oxidative stress in heart tissue

Animals were euthanized, and hearts were removed and rinsed retrogradely with a Krebs–Ringer solution (115 mM NaCl, 5 mM KCl, 1.2 mM KH_2_PO_4_, 25 mM NaHCO_3_, 1.2 mM MgSO_4_, 1.25 mM CaCl_2_ and 11 mM glucose) at the end of the study. The temperature of the perfusing solution was maintained at 37 °C. The hearts were then weighed, and cardiac tissues were homogenized on ice in chilled phosphate-buffered saline (PBS) at pH 7.4, containing 1 mM EDTA. The homogenates were centrifuged in cold saline for 10 min at 7000 rpm/min. The protein concentration of the supernatant was determined by the bovine serum albumin kit as a standard. The supernatants were used for analysis of the lipid hydroperoxide level and glutathione peroxidase (GSH-Px), superoxide dismutase (SOD), and malondialdehyde (MDA) levels. The measurements were performed by a spectrophotometer using commercial kits (Solarbio, China) according to the manufacturer’s protocols. The lipid hydroperoxide level was expressed as nmol/mg protein. The GSH-Px, SOD, and MDA levels were expressed as μmol/mg protein, nmol/mg protein, and mmol/mg protein, respectively. The expression level of NOX4 was measured by Western blotting. Mouse monoclonal anti-Nox4 (1:1000, Abcam) and horseradish peroxidase–conjugated secondary antibody (CWBIO) were used. β-Actin (1:1000, Abcam) was used as a reference. The details of this method are described below.

### Histological and immunohistochemical analysis

The heart tissues were fixed in 4% paraformaldehyde in 0.1 M phosphate buffer for 48 h, dehydrated and embedded in paraffin, sectioned at 4-μm thickness, and mounted on glass slides. Masson’s trichrome staining was used to assess the extent of fibrosis in cardiac muscle.

For antigen retrieval, the deparaffinized slides were kept in a solution of 10 mM sodium citrate (pH 6.0) for 10 min at 100 °C. The sections were then incubated in 3% hydrogen peroxide for blocking endogenous peroxidase activity and incubated with primary antibody (mouse monoclonal antibody to TGF-β1, collagen I and collagen III; Abcam) for 1.5 h, followed by corresponding secondary antibody for 2.5 h at room temperature. Subsequently, the sections were washed in PBS three times and incubated in 0.02% diamino benzidine solution for 2–8 min. After counterstaining with haematoxylin, the slides were washed briefly, mounted with resinene, and observed in the light microscope.

### Analysis of the Nrf2/ARE and TGF-β/SMAD pathway by western blotting

The Western blotting protocol was described in our previous study. Briefly, myocardial tissue was lysed in ice-cold RIPA buffer (150 of mM sodium chloride, 0.1% sodium dodecyl sulphate (SDS), 0.5% sodium deoxycholate, 1.0% NP-40, PMSF 1 mM, and 50 mM of Tris, pH 8.0) for total protein extraction. The total protein concentration was quantified by a BCA Protein Assay Kit (Medchem Express, USA). Equal amounts of protein were separated by 12% SDS–polyacrylamide gel electrophoresis (PAGE). Then, the protein was transferred from the gel to a polyvinylidene fluoride membrane. After blocking with 5% skim milk, the membrane was incubated overnight in primary antibody (Nrf2, HO-1, TGF-β1, p-Smad2, Smad2, p-Smad3, Smad3, Smad7, α-SMA 1:1000, Abcam). Bands were detected with specific horseradish peroxidase-conjugated secondary antibody (CWBIO). β-actin (1:1000, Abcam) was used as a reference of total cell protein. The density of the signal was quantified by ImageJ (version 1.8.0).

### Statistical analysis

All data were analysed using GraphPad Prism statistical software. Data were presented as mean and standard deviation (SD). For statistical evaluation, statistical differences among groups were performed by one-way analysis of variance followed by least significant difference test. *P* values less than 0.05 were considered to indicate statistically significant differences.

## Results

### Effect of empagliflozin on diabetes-related parameters

The diabetes-related parameters for mice in three groups are summarized in Table 1. Body weight was measured throughout the study, and body weight gain was calculated after 8 weeks of treatment. Because of the difference in body size between adult C57BL/6J mice and KK-Ay mice, there was a difference in body weight at the beginning of the study. Therefore, there was statistical difference in body weight after 8 weeks of feeding between the three groups. But the body weight gain decreased in the mice of the DM + EM group and was significantly affected by empagliflozin therapy in the DM + EM group. Meanwhile, heart weight and heart weight/tibial length ratio of mice were significantly different among the three groups (*P *< 0.05). However, the heart weight/tibial length ratio in the DM + EM group has no significant difference with that in the DM group (*P *< 0.05). Fasting and non-fasting blood glucose levels of mice in the DM + EM group were significantly decreased after 8 weeks of empagliflozin treatment compared with the DM group, but it was still higher than the blood glucose in the control group mice. The parameter for long-term glycaemic conditions, HbA1c, was dramatically increased in DM mice and was significantly decreased by the dose of empagliflozin. At the end of drug treatment, relative to control and DM groups, empagliflozin could significantly reduce the insulin level in DM mice. While statistical differences in TC, HDL-C, LDL-C, and TG were detected among the groups, the above four parameters were significantly increased in the DM and DM + EM groups compared with the control group (*P *< 0.05). However, there were no significant differences between the DM and DM + EM group in TC, LDL-C, and TG levels (*P *< 0.05).

**Table 1:** Weight gain, and blood and serum parameters after 8 weeks of treatment in control and diabetic mice

	Con (n = 15)	DM (n = 15)	DM + EM (n = 15)	*P* value
Body weight (g)	25.46 ± 0.70	33.61 ± 1.40*	32.17 ± 1.23*	< 0.001
Body weight gain (g)	5.32 ± 0.64	8.05 ± 0.64*	6.61 ± 0.42*^#^	< 0.001
Heart weight (mg)	130.45 ± 5.81	152.37 ± 6.26*	149.13 ± 7.87*	< 0.001
Heart weight/Tibial length (mg/mm)	7.73 ± 0.25	8.72 ± 0.32*	8.42 ± 0.27*	< 0.001
Blood glucose (mmol/L, fasting)	6.38 ± 0.27	18.83 ± 1.17*	9.27 ± 0.76*^#^	< 0.001
Blood glucose (mmol/L, non-fasting)	8.26 ± 0.38	26.39 ± 1.38*	12.51 ± 1.08*^#^	< 0.001
HbA1c (mmol/mol)	22.93 ± 2.82	94.71 ± 6.52*	39.57 ± 4.95*^#^	< 0.001
Insulin (μg/L)	1.71 ± 0.22	4.92 ± 0.43*	2.69 ± 0.51*^#^	< 0.001
TC (mg/dL)	87.59 ± 3.66	215.89 ± 14.08*	216.35 ± 15.67*	< 0.001
HDL-C (mg/dL)	32.76 ± 2.29	73.16 ± 6.51*	75.71 ± 4.71*	< 0.001
LDL-C (mg/dL)	40.59 ± 4.92	63.54 ± 6.35*	62.12 ± 5.96*	< 0.001
TG (mg/dL)	94.24 ± 6.25	314.87 ± 24.10*	310.47 ± 25.67*	< 0.001

Data are expressed as the mean ± SD

*TC* total cholesterol, *TG* triglyceride, *HDL-C* high-density lipoprotein, *LDL-C* low-density lipoprotein

*P < 0.05 vs. Con; ^#^*P* < 0.05 vs. DM

### Effect of empagliflozin on LV dimensions and functions

**Fig. 1:**
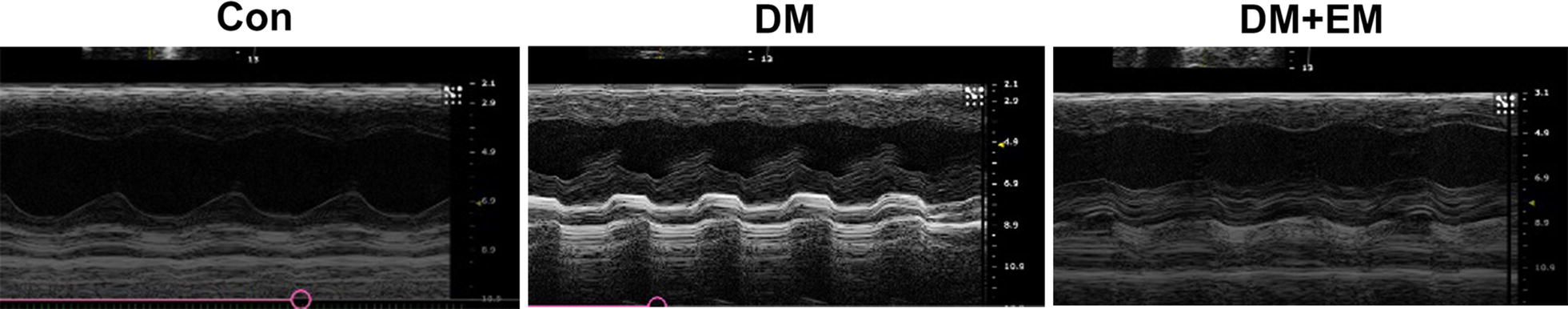
Left ventricular echocardiographic representative images

To explore whether LV remodelling and cardiac function in DM mice could be improved by empagliflozin, the cardiac parameters were measured by M-mode and Doppler echocardiographies. As shown in Fig. 1 and Table 2, the HR of DM mice was slightly lower than that of the control and DM + EM groups (*P *< 0.05) and no statistically significant difference was detected between the control and DM + EM groups (*P *> 0.05). LV mass was decreased in all of the diabetic groups compared with the control group. However, the LV mass/body weight ratio in DM mice was significantly lower than that in control and DM + EM mice (*P *< 0.05). Treatment with empagliflozin could simultaneously inhibit the reduction of LVIDd and decrease IVSd. While there were no significant differences in the values of LVIDs and IVSs among the three groups, there was obvious impairment in EF, FS, fractional area change (FAC) and E/A ratio in diabetic mice compared with the other groups. Empagliflozin treatment could largely restore EF, FS, FAC, and E/A ratio in DM mice, which were similar to those in the control group (Table 2).

**Table 2:** Echocardiographic assessment of left ventricle structural and functional data in mice

	Con (n = 15)	DM (n = 15)	DM + EM (n = 15)	*P* value
Heart rate (bpm)	473.13 ± 24.31	421.22 ± 20.35*	465.60 ± 22.82^#^	< 0.001
LVIDd (mm)	3.43 ± 0.31	2.94 ± 0.23*	3.33 ± 0.28^#^	< 0.001
LVIDs (mm)	2.33 ± 0.17	2.22 ± 0.15	2.05 ± 0.17	0.059
IVSd (mm)	1.05 ± 0.08	1.06 ± 0.06	0.94 ± 0.08*^#^	0.005
IVSs (mm)	1.35 ± 0.07	1.36 ± 0.08	1.31 ± 0.09	0.815
LV mass (mg)	92.60 ± 9.75	74.23 ± 8.53*	86.49 ± 9.12*^#^	< 0.001
LV mass/BW (mg/g)	3.65 ± 0.44	2.21 ± 0.25*	2.69 ± 0.30*^#^	< 0.001
EF (%)	70.26 ± 5.46	60.22 ± 6.62*	73.73 ± 5.79^#^	< 0.001
FS (%)	33.08 ± 3.12	23.14 ± 2.37*	37.22 ± 3.25*^#^	< 0.001
FAC (%)	70.42 ± 7.72	54.27 ± 7.49*	71.38 ± 7.69^#^	< 0.001
E/A	1.73 ± 0.24	1.22 ± 0.15*	1.70 ± 0.24^#^	< 0.001

Data are presented as the mean ± SD. P values were calculated using a one-way analysis of variance test and LSD test was used for multiple comparisons. Data are expressed as the mean ± SD

*LVIDd* LV internal diastolic diameter, *LVIDs* LV internal systolic diameter, *IVSd* interventricular septal width during end-diastole, *IVSs* systolic interventricular septal thickness, *LV* left ventricle, *BW* body weight, *EF* ejection fraction, *FS* fractional shortening, *FAC* fractional area change, *E/A* ratio between early (E)-to-late (A) diastolic mitral inflow

*P < 0.05 vs. Con; ^#^*P* < 0.05 vs. DM

### Oxidative stress in cardiac tissues

**Fig. 2:**
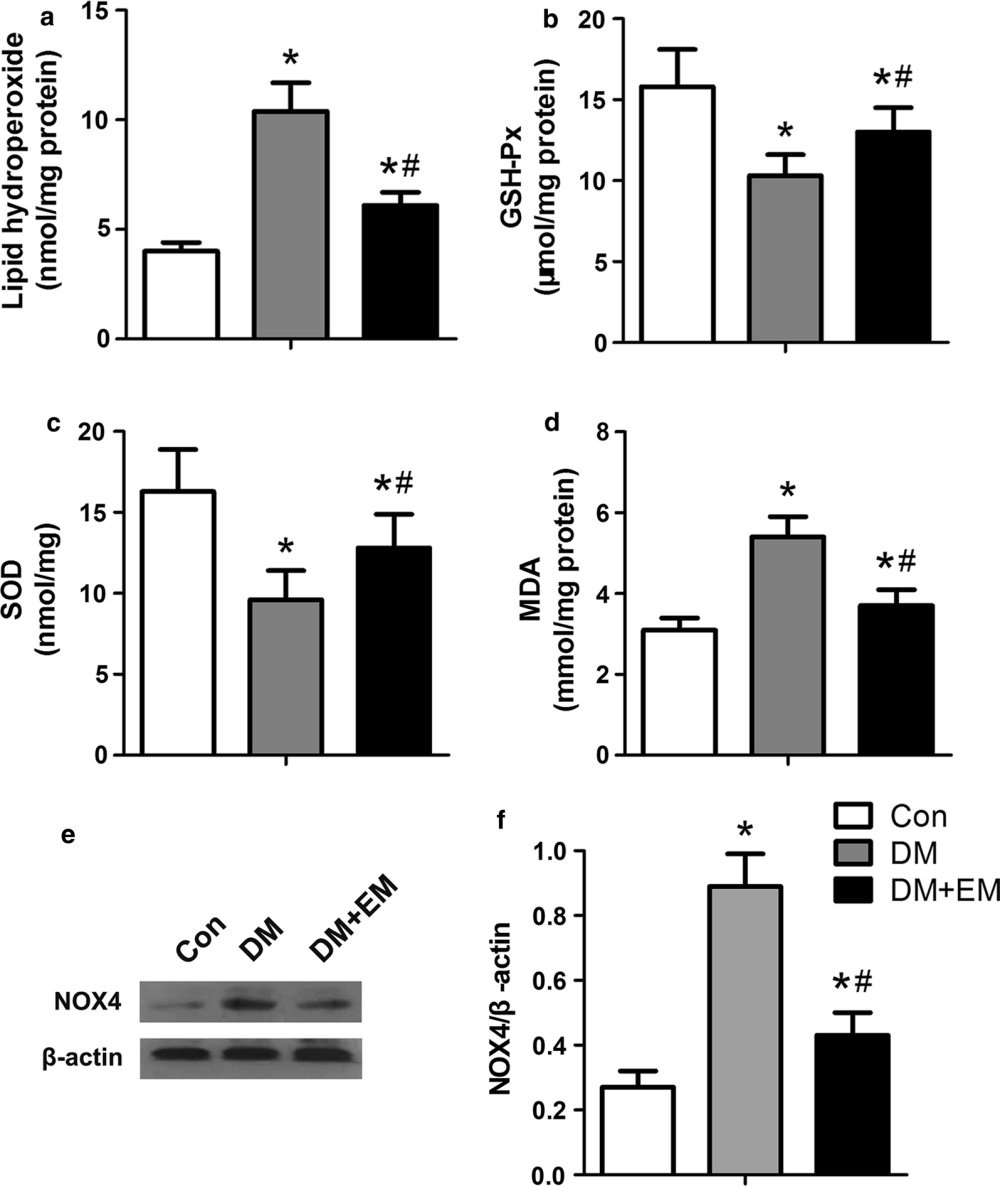
Effect of empagliflozin on oxidative stress in the cardiac tissue homogenate. Lipid hydroperoxide (**a**), glutathione peroxidase (**b**), superoxide dismutase (**c**), malondialdehyde (**d**), Western blotting analysis of NOX4 in the mice myocardium (**e**, **f**). Data are expressed as the mean ± SD. *P < 0.05 vs. Con; ^#^*P* < 0.05 vs. DM

Excessive oxidative stress is an inducer of diabetic cardiomyopathy in mice in response to high glucose levels. To determine the effect of empagliflozin on oxidative stress in diabetic mice, we measured the levels of lipid hydroperoxide, GSH-Px, SOD, and MDA in cardiac tissue. Oxidative stress parameters are shown in Fig. 2. Lipid hydroperoxide concentration and MDA level were significantly higher in DM mice than in control and DM + EM groups (*P *< 0.05), whereas the levels of SOD and GSH-Px were significantly lower in DM mice compared with diabetic mice treated with empagliflozin (*P *< 0.05). Vascular nicotinamide adenine dinucleotide phosphate (NADPH) oxidase is a major source of ROS. We tested the expression of NOX4, the major NAD(P)H oxidase isoform in cardiomyocytes, which is associated with cardiomyopathy in the diabetes model. We found that the NOX4 was greatly elevated in DM mice compared with the control group, and NOX4 expression in the DM + EM group significantly decreased compared with DM mice (*P *< 0.05). The results indicate that empagliflozin can alleviate excessive oxidative stress by elevating the level of antioxidant enzymes and reducing oxidation products in the cardiac tissue of DM mice.

### Empagliflozin treatment inhibits myocardial fibrosis in diabetic mice

**Fig. 3:**
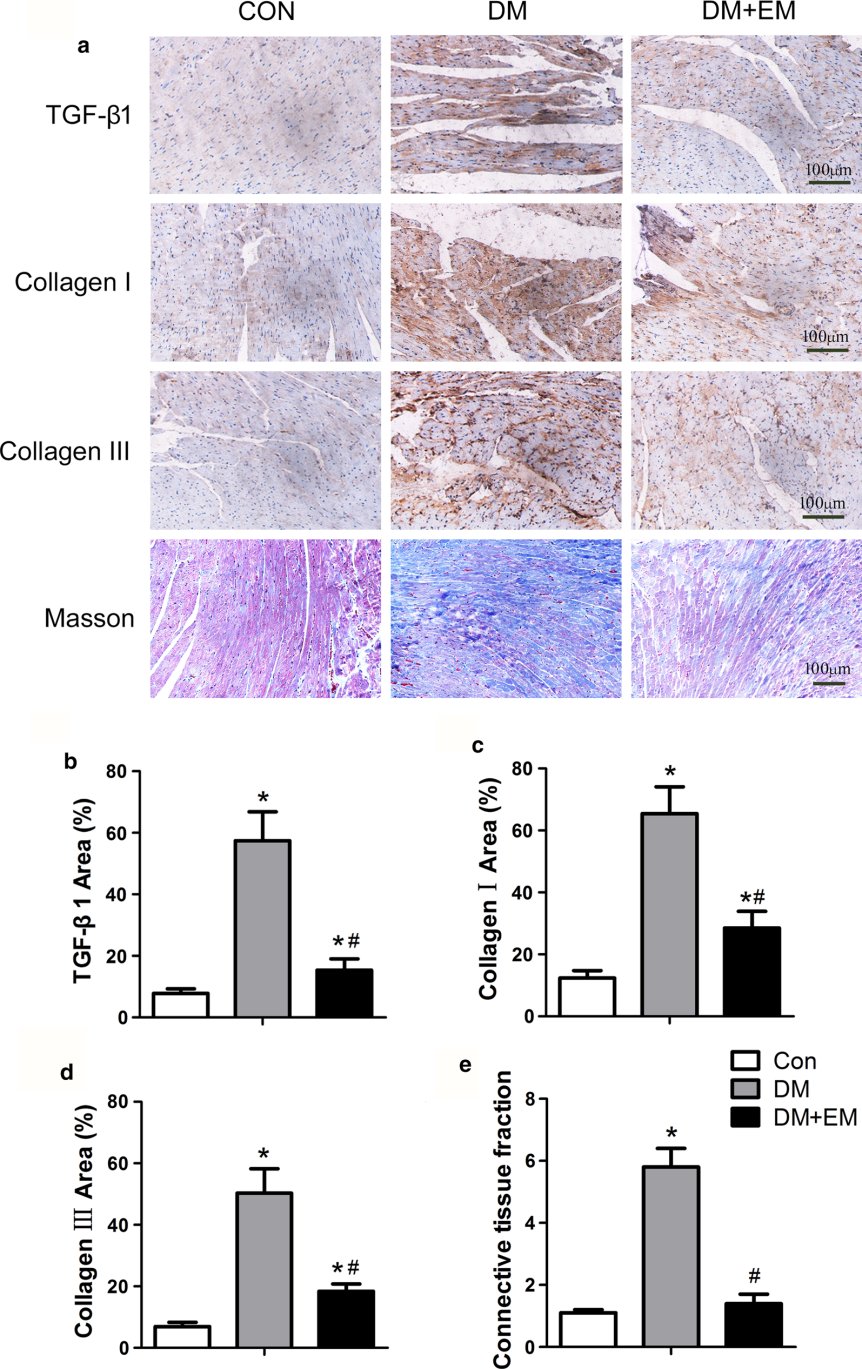
Empagliflozin suppresses matrix accumulation and myocardial fibrosis in DM mice. Immunostaining of TGF-β1, collagen I and collagen III protein expression and Masson’s trichrome staining of the myocardium (**a**). The percentages of positive areas of TGF-β1 (**b**), collagen I (**c**), collagen III (**d**) and connective tissue fraction (**e**). Data are expressed as the mean ± SD. *P < 0.05 vs. Con; ^#^*P* < 0.05 vs. DM

Immunohistochemical staining of TGF-β1 showed that brown-stained positive cells of TGF-β1 increased significantly and were distributed in the myocardial tissue in the DM group (Fig. 3a, b). Compared with the DM group, empagliflozin markedly reduced the expression of TGF-β1 by about 73.2% (*P *< 0.05). In addition, immunohistochemistry analysis of the expression levels of collagen I and collagen III proteins revealed significant differences among three groups (all *P *< 0.05). The positive percentages of collagen I and collagen III decreased dramatically in the DM + EM group (28.5% ± 5.4% and 18.4% ± 2.4%, respectively) as compared with the DM group (65.4% ± 8.7% and 50.3% ± 7.9%, respectively; all *P* < 0.05, Fig. 3c, d). To further evaluate the degree of myocardial fibrosis in mice, we used the Masson’s trichrome stain method. Connective tissue is stained blue, nuclei are stained dark purple, and cytoplasm is stained red. The analysis of the Masson’s trichrome stain pictures revealed that there was a significant difference in the median cardiac connective tissue fraction among the three groups (*P* < 0.05). The DM group (5.8 ± 0.6) had the highest connective tissue fraction when compared with the control group (1.1 ± 0.1) and the DM + EM group (1.4 ± 0.3; all *P* < 0.05, Fig. 3e). However, there was no significant difference in cardiac connective tissue fraction between the control and DM + EM groups. These results suggest that empagliflozin can effectively inhibit myocardial fibrosis in diabetic mice.

### Effect of empagliflozin on Nrf2/ARE and TGF-β/SMAD pathway in mouse heart

**Fig. 4:**
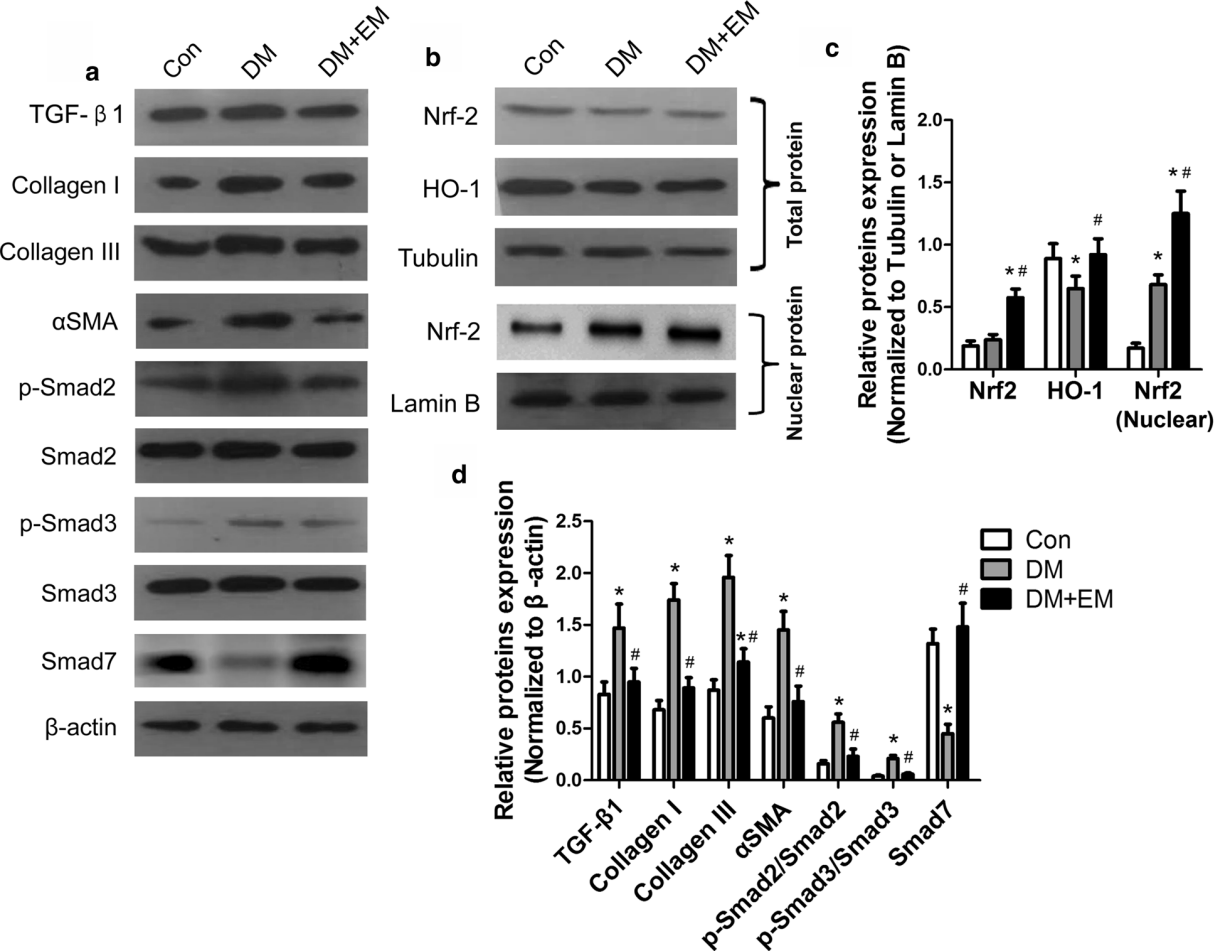
Effect of empagliflozin on Nrf2/ARE and TGF-β/SMAD pathway in vivo.** a** Western blot analysis for the expression of TGF-β/SMAD pathway was performed on protein isolated from the hearts of the three groups.** b** Western blot analysis for the expression of Nrf2/ARE pathway of heart tissue in mice.** c** The relative protein levels were calculated and Lamin B was used as an internal control.** d** The relative protein levels were calculated and β-actin was used as an internal control. Data are expressed as the mean ± SD. *P < 0.05 vs. Con; ^#^*P* < 0.05 vs. DM

The Nrf2/ARE pathway is an essential signalling pathway in regulating oxidative stress. Previous results showed an increase in oxidative stress in the vascular tissues of patients with DM. Based on the above results, we confirmed that the oxidative stress markers of lipid hydroperoxide and MDA were greatly elevated in cardiac tissues of DM mice compared with DM + EM mice. Therefore, we will further explore whether empagliflozin caused the activation of the Nrf2/ARE pathway and downregulation of the oxidative stress in mouse heart tissue. Western blot analysis of Nrf2 and HO-1 protein expression levels showed that Nrf2 and HO-1 levels of total protein or nuclear protein in the DM + EM group increased dramatically compared with those in the DM group (P < 0.05) (Fig. 4b, c). These results indicate that empagliflozin can promote the nuclear translocation of Nrf2 and reduce the oxidative stress levels in DM mice.

Because TGF-β/Smad signalling is considered a the major factor in the regulation fibrosis, we detected TGF-β/SMAD signalling in the three groups using Western blot. TGF-β/SMAD signalling was highly activated in the heart tissue of diabetic mice, as revealed by a significant upregulation of TGF-β1, p-Smad2, and p-Smad3 and higher levels of collagen I, collagen III, and αSMA. Treatment with empagliflozin significantly suppressed a marked upregulation of TGF-β1, p-Smad2, p-Smad3, collagen I, collagen III, and αSMA in the heart tissue of diabetic mice (Fig. 4a, d). Smad7, a negative inhibitor of TGF-β/SMAD signal, was decreased in the DM group and greatly increased with empagliflozin treatment. These data suggest that empagliflozin was effective in inhibiting the TGF-β/Smad pathway of DM mice.

## Discussion

Pharmacological intervention inhibiting SGLT2, which mediates 90% of renal glucose reabsorption, has been proposed as a novel therapeutic strategy for diabetes. Empagliflozin, a selective inhibitor of SGLT2, represents a new class of drugs for treatment of type 2 DM by increasing glucose excretion in urine and acting independently of insulin. In addition to its excellent glycaemic effect, there is much evidence of the extra-glycemic benefits of empagliflozin, such as weight loss; decrease in blood pressure, uric acid levels, and TGs; increase in haematocrit and blood viscosity; and slowdown of kidney disease progression [17, 18]. In this study, we evaluated the influence of the SGLT2 inhibitor empagliflozin on cardiac oxidative stress and remodeling in mice with type 2 DM. The key findings of this study were that treatment with empagliflozin significantly ameliorated myocardial fibrosis and oxidative stress in type 2 diabetic mice. We confirmed that empagliflozin could effectively control blood glucose and reduce insulin release. In addition its role in good glycemic control, empagliflozin also significantly lowers blood cholesterol and TG levels in diabetic mice. Furthermore, these beneficial effects of empagliflozin were associated with the obvious mitigation of LV functional and structural impairment induced by DM. Moreover, empagliflozin ameliorated cardiac oxidative stress and fibrosis and decreased extracellular matrix proteins such as type I collagen, type III collagen, and αSMA deposits associated with activating Nrf2/ARE signalling and suppressing TGF-β/SMAD signalling. This experimental evidence provides support for empagliflozin as a potentially promising therapeutic drug for cardiomyopathy and cardiovascular complications in type 2 diabetes.

Phlorizin, a natural compound found in the root bark of apple, has been used as a potential pharmaceutical treatment for type 2 diabetes [19]. Given that phlorizin is a competitive inhibitor of SGLT1 and SGLT2, researchers have developed a highly selective inhibitor of SGLT2 empagliflozin for the treatment of type 2 diabetes. Many animal experiments and clinical applications have also validated its good ability for lowering blood glucose [20, 21]. The type 2 diabetic KK-Ay mice model used in this study was characterized by chronic hyperglycaemia, dysregulation of haemoglobin glycation (HbA1c), and weight loss. In the long-term experiment of the present study, blood glucose and insulin levels were lower and body weight gain was greater in empagliflozin-treated DM mice than in DM mice. Accumulating clinical data indicate that empagliflozin reduces body weight and glucose levels in an insulin-independent pattern in type 2 diabetic patients [22–24]. Interestingly, our results suggest that empagliflozin exerted glycemic control effects but did not improve the serum lipid profile in diabetic mice. Another previous study showed that empagliflozin increases ketone production and LDL-C levels by reducing intestinal cholesterol absorption in hamsters with diet-induced dyslipidaemia and diabetes [25]. However, in that clinical trial that no changes in LDL-C or TGs were observed between DM patients and DM patients treated with empagliflozin [26]. Therefore, the mechanism of the effect of empagliflozin on blood lipids needs further exploration.

Oxidative stress is defined as a state in which cell injury and generation of excessive ROS in vivo exceed the cells’ inherent capacity of antioxidant defences thus damaging proteins, lipids, and DNA. Previous experimental and clinical studies have confirmed that diabetes can cause elevated levels of ROS in vivo [27, 28]. It is generally accepted that hyperglycaemia-induced ROS is due to cell dysfunction via NADPH oxidase and mitochondrial electron transport chain. Glucose accumulation promotes ROS, production of advanced glycation end products, and elevated levels of O-GlcNAcylated protein [29]. A large amount of ROS also accumulates in the myocardial tissue in diabetes. There are several sources of ROS in diabetic myocardium, such as increased activity of NADPH oxidase, leakage of the mitochondrial electron transport chain, and uncoupling of nitric oxide synthase. Here, we found that oxidative stress was greatly increased in DM mice. Increased NADPH oxidase activity was also observed in the myocardium and vascular tissues in the type 2 diabetes animal model [30, 31]. This is consistent with our findings. Meanwhile, we also found that empagliflozin can significantly downregulate the level of NOX4 in the myocardial tissue of diabetic rats, namely, by reducing NADPH oxidase activity. Therefore, empagliflozin may alleviate diabetic cardiomyopathy via inhibition of oxidative stress generated by NADPH oxidases. In patients with diabetes, both the activity of the NADPH oxidase system and the levels of NADPH oxidase protein subunits were significantly elevated [32]. In the present study, we observed that empagliflozin had a negative influence on diabetes-induced oxidative stress in the myocardium of mice. In addition, we explored the potential mechanism and found that empagliflozin may promote Nrf2 translocation to the nucleus and activate the Nrf2/ARE signalling to inhibit oxidative stress in the myocardium. Previous studies have implicated Nrf2 deletion in the impairment of cellular stress, glucose tolerance, and exacerbation of hyperglycaemia in a diabetic animal model [33, 34]. The importance and role of Nrf2 and its downstream elements have been illuminated in previous studies and include their ability to preserve endothelial cell function, regulate blood pressure, and protect the myocardium [35]. In particular, upregulation of HO-1 improves LVEF and inhibits myocardial remodelling in diabetic mice [36]. Dapagliflozin has an important antioxidant-like cardio-protective effect via Zn^2+^-transporters, matrix metalloproteinases, and oxidative stress [37]. In view of the close relationship between Nrf2 and oxidative stress levels in diabetic cardiomyopathy, empagliflozin is a promising drug for reducing oxidative stress in myocardium by targeting Nrf2 signalling.

Extensive clinical evidence has documented the presence of myocardial fibrosis in patients with diabetes [9, 38]. Many histopathological studies have also confirmed that cardiac fibrosis caused by diabetes occurs independently of hypertension or coronary atherosclerosis [39]. In our study, all diabetic mice showed significant myocardial fibrosis in view of biochemical and histopathological changes. In addition, we also found that diabetic mice had structural and functional alterations at the level of the myocardium. In the present study, empagliflozin treatment for 8 weeks successfully preserved cardiac systolic function. There was a significant difference in the E/A ratio, a marker of LV diastolic function, or collagen I, collagen III, and cardiac connective tissue fraction between the DM + EM and DM groups. In addition, other parameters mentioned above that were associated with LV structure and function were comparable between the DM + EM and DM groups. These findings suggest that empagliflozin treatment appears to effective in rescuing diabetic cardiomyopathy. A previous study demonstrated that empagliflozin was capable of improving cardiac microvascular perfusion, microvessel density, endothelial-dependent relaxation, cardiac microvascular endothelial cell survival and diabetic myocardial structure and function [40]. Dapagliflozin treatment improves endothelial dysfunction, vascular smooth muscle dysfunction and arterial stiffness in type 2 diabetic mice or patients with type 2 DM [41–43]. Empagliflozin reduces glucotoxicity, prevents the development of endothelial dysfunction and reduces oxidative stress in Zucker diabetic fatty rats [44]. The TGF-β/SMAD pathway appears to be involved in the process of collagen production in a variety of cell types and organs. Indeed, many studies have confirmed that TGF-β1 strongly contributes to fibrotic disorders in heart disease [45]. In our study, it was shown that empagliflozin downregulated the expression of TGF-β1 and reduced the ratios of p-Smad2/Smad2 and p-Smad3/Smad3 in DM mice. In addition, the negative inhibitor of the TGF-β/SMAD pathway, Smad7, was significantly elevated with empagliflozin treatment. The mechanism of empagliflozin on diabetes-induced myocardial fibrosis may improve LV function by reducing collagen I, collagen III, and αSMA deposition in myocardial tissue. Empagliflozin has been recently shown to ameliorate cardiac hypertrophy and fibrosis in rats with prediabetic metabolic syndrome, thus supporting our speculation [46]. In addition, other studies have suggested that SGLT2 inhibitors reduced hyperfiltration and decreased inflammatory and renal fibrosis [47–49]. Moreover, dapagliflozin attenuated cardiac fibrosis by regulating the macrophage polarization via STAT3 signaling in infarcted rat hearts [50]. In addition to the effect of lowering blood glucose on improving diabetic cardiomyopathy, SGLT2 inhibitors may also improve diabetic cardiomyopathy by improving ketone levels and utilization in the body and regulating uric acid levels. SGLT2 inhibitors can cause a slight increase in levels of ketone in the body. Ferrannini et al. hypothesized that empagliflozin may exhibit some of its beneficial effects through a shift in myocardial metabolism toward an energy-efficient use of ketone bodies, which may improve myocardial work efficiency and function [51]. SGLT2 inhibitors promotes blood glucose excretion by inhibiting reabsorption of glucose from glomerular filtrate and also increases uric acid excretion, which are cardioprotective and associated with cardiovascular complications and congestive heart failure [52, 53]. In an experiment using a prediabetic metabolic syndrome rats model, empagliflozin was also shown to reduce cardiac hypertrophy and myocardial fibrosis by inhibiting oxidative stress and inflammatory responses [46]. In line with our findings, this result indicates that empagliflozin can achieve the same cardioprotective effects in the context of obesity. In addition, it was found that empagliflozin can ameliorate cardiovascular injury, vascular dysfunction, and cognitive decline in the context of a mouse model of type 2 diabetes with spontaneous mutation of the leptin receptor [54, 55]. Habibi et al. observed interventional improvement of empagliflozin in fibrosis and diastolic dysfunction in the absence of significant reductions in myocardial oxidative/nitrosative stress using 3-nitrotyrosine immunostaining for determination of oxidative stress [55]. The discrepancy between our findings and those findings of other studies may be because the 3 nitrotyrosine reflects partial oxidative stress and its level is also related to the concentration of tyrosine in plasma. Therefore, various indicators are needed to evaluate the level of tissue oxidative stress. Meanwhile, we have explored the underlying mechanisms of empagliflozin in the prevention and treatment of diabetic cardiomyopathy. Furthermore, we need to further explore the mechanism of the effect of empagliflozin in the type 2 diabetic mice model with different genetic backgrounds. Thus, these experimental data indicate the therapeutic promises of empagliflozin in treating myocardial fibrosis, and the underlying mechanism deserves further investigation.

## Conclusions

In summary, the SGLT2 inhibitor empagliflozin ameliorates myocardial fibrosis partly through inhibition of collagen formation and deposition via the classical TGF-β/Smad pathway and decreases oxidative stress via promoting Nrf2 translocation to the nucleus and activating Nrf2/ARE signalling in the type 2 diabetic KK-Ay mice model. In addition, 8 weeks of empagliflozin treatment rescues the LV structure and function in diabetic mice. Therefore, empagliflozin is a promising agent in the prevention and treatment of diabetic cardiomyopathy. However, more clinical data are needed to verify the safety and efficacy of empagliflozin in treating patients with diabetic cardiomyopathy, and its potential mechanism also needs further exploration.

## Abbreviations

SGLT2: sodium-glucose cotransporter 2
DM: diabetes mellitus
TGF-β: transforming growth factor-β
ROS: reactive oxygen species
NAD+: nicotinamide adenine dinucleotide
HR: heart rate
LVIDD: left ventricular internal dimension in diastole
LVIDS: left ventricular internal dimension in systole
IVSs: interventricular septal thickness in systole
IVSd: interventricular septal thickness in diastole
LVPWs: left ventricular posterior wall thickness in systole
LVPWd: left ventricular posterior wall thickness in diastole
FS: fractional shortening
EF: ejection fraction
TC: total cholesterol
TG: triglyceride
LDL-C: low-density lipoprotein cholesterol
HDL-C: high-density lipoprotein cholesterol
ELISA: enzyme-linked immunosorbent assay
PBS: phosphate buffered saline
GSH-Px: glutathione peroxidase
SOD: superoxide dismutase
MDA: malondialdehyde
SDS: sodium dodecyl sulphate
PAGE: polyacrylamide gel electrophoresis
PVDF: polyvinylidene fluoride
SD: standard deviation
HbA1c: haemoglobin glycation
NADPH: nicotinamide adenine dinucleotide phosphate
